# Authentication of commercial processed *Glehniae Radix* (*Beishashen*) by DNA barcodes

**DOI:** 10.1186/s13020-015-0071-8

**Published:** 2015-11-30

**Authors:** Xunzhi Zhu, Yuxi Zhang, Xia Liu, Dianyun Hou, Ting Gao

**Affiliations:** Key Laboratory of Plant Biotechnology in Universities of Shandong Province, College of Life Sciences, Qingdao Agricultural University, Qingdao, Shandong China; College of Biotechnology, Jiangsu University of Science and Technology, Zhenjiang, Jiangsu China; School of Chemistry, Chemical Engineering and Life Science, Wuhan University of Technology, Wuhan, Hubei China; Agricultural College, Henan University of Science and Technology, Luoyang, Henan China; The National Engineering Laboratory for Breeding of Endangered Medicinal Materials, Institute of Medicinal Plant Development, Chinese Academy of Medical Sciences and Peking Union Medical College, Beijing, China

## Abstract

**Background:**

The radix of *Glehnia littoralis* Fr. Schmidt ex Miq. (*Beishashen*), is often misidentified and adultered in Chinese medicine. Its seven common adulterants include *Chuanminshen violaceum* Sheh et Shan (*Chuanmingshen*), *Changium smyrnioides* Wolff (*Mingdangshen*), *Sphallerocarpus gracilis* (Bess.) K.-Pol. (*Miguoqin*), *Adenophora polyantha* Nakai (*Shishashen*), *Silene tatarinowii* Regel (*Shishengyingzicao*), *Adenophora tetraphylla* (Thunb.) Fisch (*Lunyeshashen*) and *Adenophora stricta* Miq. (*Shashen*). This study aims to evaluate the feasibility of the second internal transcribed spacer (ITS2) DNA barcoding to discriminate between *Glehniae Radix* and its common adulterants.

**Methods:**

In this study, we collected 46 samples of *G. littoralis* and 59 samples of its seven common adulterants. Genomic DNA sequences were extracted from samples, including original plants and commercially processed crude drugs. The ITS2 of the ribosomal DNA sequences were amplified and sequenced bi-directionally. The sequences were assembled by CodonCode Aligner 3.5.7. The descriptive data analysis was conducted and neighbor-joining (NJ) phylogenetic tree was constructed by MEGA 5.0 in accordance with the kimura 2 -parameter (K2P) model. The identification efficiency was evaluated based on the BLAST1 methods. The ITS2 secondary structures were predicted and compared between *Glehniae Radix* and its adulterants by the ITS2 database.

**Results:**

As the 46 ITS2 sequences of *G. littoralis* were identical to each other, the identification efficiency of the ITS2 region was 100 %. A NJ tree based on the ITS2 sequences, and the predicted secondary structures of ITS2, distinguished *Glehniae Radix* from its adulterants.

**Conclusion:**

DNA barcoding based on ITS2 distinguished commercial processed *Glehniae Radix* from common herbal adulterants.

**Electronic supplementary material:**

The online version of this article (doi:10.1186/s13020-015-0071-8) contains supplementary material, which is available to authorized users.

## Background

The radix of *Glehnia littoralis* (*beishashen*) is used as an antitussive, mucolytic, antibacterial, antiphlogistic and immune response enhancer in Chinese medicine (CM) [1]. It is also used as a diaphoretic, antipyretic and analgesic in Japan [2]. *Glehniae Radix* is listed in the Japanese and Chinese Pharmacopoeia and is widely recognized as a nutritional and healthy food [3–6].

Due to the high market demand, overexploitation of *Glehniae Radix* already threatened the existence of this wild species. And it was protected now by China Plants Red Data Book [7]. However, the scarcity of this wild species has resulted in frequent fraudulent adulteration and substitution of *G. littoralis* with the species *Chuanminshen violaceum* Sheh et Shan (*Chuanmingshen*), *Changium smyrnioides* Wolff (*Mingdangshen*), *Sphallerocarpus gracilis* (Bess.) K.-Pol. (*Miguoqin*), *Adenophora polyantha* Nakai (*Shishashen*) and *Silene tatarinowii* Regel *(Shishengyingzicao*), because of their similar appearances [8, 9]. Due to their similar Chinese names, *Adenophorae Radix* is also easily confused with *Glehniae Radix* in clinical use [10]. The botanical origins of *Adenophorae Radix* are two species in the family Campanulaceae, *Adenophora tetraphylla* (Thunb.) Fisch (Lunyeshashen) and *Adenophora stricta* Miq. (*Shashen*).

The biologically active compounds of these adulterants are significantly distinct from those of *Glehniae Radix*. However these adulterants do not contain these biologically active compounds coumarins, coumarin glyeosides and polyacetylenes as *Glehniae Radix* [11]. The identification of *Glehniae Radix* and its adulterants has been based on morphological and microscopic observation [8, 9], while molecular identification has been rarely used [10, 12]. However, the assessment procedures are affected by environmental factors and often produce ambiguous results [13].

The DNA barcoding technique uses standard genomic regions to discriminate species [14–16], and this method provides consistent and reliable results regardless of the age, plant part or environmental factors of the sample [17]. Because of its speed and accuracy, DNA barcoding has gained attention in CM identification [12]. Although the Barcode of Life Plant Working Group (BOL) recommends the sequence combination *rbcL* + *matK* for barcoding [16], other genomic regions such as nuclear ITS (Internal Transcibed Spacer) may also be useful for medicinal material identification [17]. The ITS2 DNA sequence was suggested as a universal (medicinal) plant barcode [18–20]. The China Plant BOL Group (CBOL) has also suggested that ITS/ITS2 should be incorporated into the core barcode for seed plants [21]. The ITS2 region has been successfully applied to identify diverse medicinal plants and herbal materials [18, 22–26]. However, the ITS2 barcode was more likely to be affected by genetic anomalies, such as gene multiplication, pseudogenes and introgression [20].

For our study, we would like to identify commercial processed *Glehniae Radix* and its adulterants from a large pool of samples by the ITS2 sequences. This study aims to evaluate the suitability and feasibility of ITS2 barcode to accurately discriminate between *Glehniae Radix* and its adulterants, particularly the sequence divergences and differentiation powers of the ITS2 region.

## Methods

### Sample collection

Seven original plant samples (dried leaves or dried roots prepared from plants) and 36 commercially processed crude drug samples belonging to *G. littoralis* were collected from a large geographical area in China, including Hebei, Shandong and Inner Mongolia (Table 1). We also gathered 16 samples belonging to seven common adulterant species of *Glehniae Radix*: *C. smyrnioides*, *C. violaceum*, *A. polyantha*, *A. tetraphylla*, *A. stricta, S. gracilis* and *S. tatarinowii*. All samples were identified by Associate Professor Hongxiao Yang (College of Resources and Environment, Qingdao Agricultural University, Qingdao, China) by microscopic and morphological identification [1, 27]. Voucher specimens (Table 1) were deposited in the Herbarium of Qingdao Agricultural University. Silica gel-dried leaves or roots from individual plants were collected and commercially available crude preparations of *Glehniae Radix* and its adulterants were purchased from pharmacies. Subsequently, three ITS2 sequences of *G. littoralis* and 43 ITS2 sequences of its seven adulterants were all downloaded from GenBank for further analysis (Table 1).

**Table 1:** Plant material samples used in the study

Scientific name	Voucher no.	Taxon (sampling part)	GenBank accession no.	Time of collection	Collection place
*Glehnia littoralis*	PS001MT01	Crude drug	KF010586	Mar., 2013	Deyang, Sichuan
	PS001MT02	Crude drug	KF010588	Mar., 2013	–, Hebei
	PS001MT03	Crude drug	KF010587	Mar., 2013	Qingdao, Shandong
	PS001MT04	Dried leaf	–	Mar., 2013	Qingdao, Shandong
	PS001MT05	Crude drug	–	Mar., 2013	–, Hebei
	PS001MT06	Crude drug	–	Mar., 2013	–, Inner Mongolia
	PS001MT07	Crude drug	–	Mar., 2013	–, Shandong
	PS001MT08	Crude drug	–	Mar., 2013	Ningbo, Zhejiang
	PS001MT09	Crude drug	–	Mar.,2013	–, Hebei
	PS001MT10	Crude drug	–	Mar., 2013	Shuozhou, Shanxi
	PS001MT11	Crude drug	–	Mar., 2013	–, Hebei
	PS001MT12	Crude drug	–	Mar., 2013	Shanghai
	PS001MT13	Crude drug	–	Mar., 2013	–, Hebei
	PS001MT14	Crude drug	–	Mar., 2013	Laiyang, Shandong
	PS001MT15	Crude drug	–	Mar., 2013	–, Hebei
	PS001MT16	Crude drug	–	Mar., 2013	–, Anhui
	PS001MT17	Crude drug	–	Mar., 2013	Tianjin
	PS001MT18	Dried leaf	–	Mar., 2013	Qingdao, Shandong
	PS001MT19	Dried leaf	–	Mar., 2013	Qingdao, Shandong
	PS001MT20	Crude drug	–	Mar., 2013	Qingdao, Shandong
	PS001MT21	Crude drug	–	Mar., 2013	Guangzhou, Guangdong
	PS001MT22	Crude drug	–	Mar., 2013	Guangzhou, Guangdong
	PS001MT23	Crude drug	–	Mar., 2013	Chengdu, Sichuan
	PS001MT24	Crude drug	–	Mar., 2013	–, Sichuan
	PS001MT25	Crude drug	–	Mar., 2013	–, Hebei
	PS001MT26	Crude drug	–	Mar., 2013	–, Shandong
	PS001MT27	Dried root	–	Mar., 2013	Yantai, Shandong
	PS001MT28	Dried root	–	Mar., 2013	Yantai, Shandong
	PS001MT29	Dried root	–	Mar., 2013	Yantai, Shandong
	PS001MT30	Dried root	–	Mar., 2013	Yantai, Shandong
	PS001MT31	Crude drug	–	Mar., 2013	Anguo, Hebei
	PS001MT32	Crude drug	–	Mar., 2013	Anguo, Hebei
	PS001MT33	Crude drug	–	Mar., 2013	Anguo, Hebei
	PS001MT34	Crude drug	–	Mar., 2013	Bozhou, Anhui
	PS001MT35	Crude drug	–	Mar., 2013	Bozhou, Anhui
	PS001MT36	Crude drug	–	Mar., 2013	Wuhan, Hubei
	PS001MT37	Crude drug	–	Mar., 2013	Chengdu, Sichuan
	PS001MT38	Crude drug	–	Mar., 2013	Bozhou, Anhui
	PS001MT39	Crude drug	–	Mar., 2013	Bozhou, Anhui
	PS001MT40	Crude drug	–	Mar., 2013	Anguo, Hebei
	PS001MT41	Crude drug	–	Mar., 2013	Anguo, Hebei
	PS001MT42	Crude drug	–	Mar., 2013	Anguo, Hebei
	PS001MT43	Crude drug	–	Mar., 2014	Guangzhou, Guangdong
	PS001MT44	–	GU395183	–	GenBank
	PS001MT45	–	FJ593179	–	GenBank
	PS001MT46	–	EU164928	–	GenBank
*Adenophora tetraphylla*	PS002MT01	Dried root	KM191311	Sep, 2013	Nanyang, Henan
	PS002MT02	Dried root	KM191312	Sep, 2013	Pingxiang, Jiangxi
	PS002MT03	Dried root	KM191313	Jun., 2014	Qingdao, Shandong
	PS002MT04	Dried root	KM191314	Jun., 2014	Qingdao, Shandong
	PS002MT05	Dried root	KM191315	Jun., 2014	Qingdao, Shandong
	PS002MT06	–	AY548194	–	GenBank
	PS002MT07	–	EU591967	–	GenBank
	PS002MT08– PS002MT09	–	KF175313–KF175314	–	GenBank
*Adenophora stricta*	PS003MT01	Dried root	KM191316	Mar., 2014	Shanghai
	PS003MT02	Dried root	KM191317	Mar., 2014	Shanghai
	PS003MT03	Dried root	KM191318	Mar., 2014	Shanghai
	PS003MT04	Dried root	KM191319	Mar., 2014	Shanghai
	PS003MT05	Dried root	KM191320	Mar., 2014	Shanghai
	PS003MT06	–	HQ704529	–	GenBank
	PS003MT07	–	AF090713	–	GenBank
*Adenophora polyantha*	PS004MT01	Dried root	KM233191	Jun., 2014	Qingdao, Shandong
	PS004MT02	Dried root	KM233192	Jun., 2014	Qingdao, Shandong
	PS004MT03- PS004MT06	–	KF175317- KF175320	–	GenBank
	PS004MT07	–	HQ704524	–	GenBank
*Silene tatarinowii*	PS005MT01 PS005MT02	Dried leaf –	KM191321 FJ384025	Mar., 2014 –	Beijing GenBank
*Changium smyrnioides*	PS006MT01	–	DQ517340	–	GenBank
	PS006MT02– PS006MT19	–	HQ185237- HQ185254	–	GenBank
	PS006MT20– PS006MT24	–	EU515301- EU515305	–	GenBank
	PS006MT25– PS006MT26	–	KF573823- KF573824	–	GenBank
*Sphallerocarpusgracilis*	PS007MT01	Dried leaf	KM191322	Mar., 2014	Beijing
	PS007MT02	–	AF073678	–	GenBank
*Chuanminshen violaceum*	PS008MT01	Crude drug	GQ434691	Mar., 2008	Jintang, Sichuan
	PS008MT02	Crude drug	KM191323	Mar., 2014	Chengdu, Sichuan
	PS008MT03 PS008MT04 PS008MT05 PS008MT06	– – – –	HQ185255 HQ185256 EU515306 FJ385040	– – – –	GenBank GenBank GenBank GenBank

The GenBank synonym name of *Adenophora tetraphylla* is *Adenophora triphylla*

### DNA extraction, amplification and sequencing

Genomic DNA was extracted from the silica gel-dried leaves, dried roots and crude drugs by the plant Genomic DNA Kit (Tiangen BioTech, Beijing, China). DNA extraction from crude drugs required the following improvements. After wiping the treated surface of the samples with 75 % ethanol (Sigma-Aldrich, USA) approximately 150 mg of interior material was obtained. Polyvinyl pyrrolidone (PVP)-30 powder (10 %, Sigma-Aldrich, USA) was added and the material was quickly ground into powder in liquid nitrogen. The process step below was repeated three times. Cold nuclear separation liquid (1 mL; 10 mmol/L Tris–HCl; pH 8.0) (Sigma-Aldrich, USA), 0.3 mol/L saccharose liquid (Sigma-Aldrich, USA), 0.4 % β-mercaptoethanol (Sigma-Aldrich, USA) was added to the powder. The mixture was allowed to stand for 2 min and then centrifuged (Eppendorf, Hamburg, Germany) at 13,500×*g* for 4 min at 4 °C. The supernatant was then discarded. Next, the precipitate was supplemented with GP1 and incubated overnight in a 65 °C water bath. GP2 was replaced with isopropyl alcohol (Sigma-Aldrich, USA). Further procedures were conducted according to the manufacturer’s instructions. We then performed a PCR amplification of ITS2 by the same primers and PCR conditions as used in previous studies [18]. The extracted DNA and PCR products were examined by 1.0 % agarose gel electrophoresis and scanned by spectrophotometer measurement (Bio-Rad, CA, USA). The purified PCR products were sequenced in both directions on a 3730XL sequencer (Invitrogen BioTech Co. Ltd., Beijing, China).

### Data analysis

Consensus and contiguous sequences were generated by a CodonCode Aligner V3.5.7 (CodonCode Co., MA, USA). Then, the ITS2 spacer sequences were obtained after removal of both the 5.8S and 28S sections of the sequences based on Hidden Markov models [28]. The obtained ITS2 sequences were shown to be reliable by the BLAST1 method. Subsequently, K2P genetic distances were calculated by MEGA 5.0 software (Arizona State University, Arizona, USA) [29], and a NJ tree was constructed based on the ITS2 sequences, with 1000 bootstrap replicates. The ITS2 species determination power was explored by the BLAST1 method, which is based on the best hit of the query sequence and an E-value for the match of less than a cutoff value, as described previously [18]. The secondary structures of the ITS2 sequences were predicted according to an online ITS2 database [30].

## Results

### DNA extraction and the efficiency of PCR amplification

The genomic DNA extracted from the root samples were extensively degraded and produced faint, diffuse bands upon DNA gel electrophoresis. The success rate for the ITS2 PCR amplification from all the samples was at 100 % (Additional file 1). Moreover, high-quality trace files were obtained for the sequenced ITS2 regions.

### Sequence and inter^−^/intra^−^ specific variation analysis

The intraspecific variation among the 43 samples of *Glehniae Radix* collected from different localities was not detected (including two partial sequences, GU395183 and AY146915). The ITS2 sequences generated in this study were identical to those from GenBank. We also found that the ITS2 regions of the commercial *Glehniae Radix* and the original plants belonged to the same haplotype. The length of these sequences was 228 bp after intraspecific alignment, and the GC content was 58.6 %. The sequence possessed a poly(A) structure at position 94–98 bp.

The *Glehniae Radix* ITS2 DNA sequences diverged considerably from those of its adulterants, which varied from 202 to 266 bp in length. The average GC content was 56.0 % in all taxa. The length of the ITS2 sequences was 275 bp after interspecific alignment, with 189 bp variable sites (68.7 %). The maximum interspecies variation was 0.987 (belonging to *S. tatarinowii*), whereas the minimum interspecies divergence was 0.248 (belonging to *S. gracilis*). The average interspecific distance was 0.468, which was greater than the *Glehniae Radix* intraspecific distance of 0.000 (Table 2). The genetic relationships between *Glehniae Radix* and its adulterants within the same family (Umbelliferae), *S. gracilis*, *C. violaceum, and C. smyrnioides,* were closer than between *Glehniae Radix* and other adulterants.

**Table 2:** Sequence characteristics of ITS2 for *G. littoralis* and its adulterants

Sequence characteristics	
Amplification efficiency of *G. littoralis*	100 %
Sequencing efficiency of *G. littoralis*	100 %
Length of *G. littoralis*	228 bp
Amplification efficiency of all taxa	100 %
Sequencing efficiency of all taxa	100 %
Length of all taxa	202–266 bp
Aligned length	275 bp
GC content range in *G. littoralis*	58.6 %
GC content range in all taxa, (mean GC content in all taxa)	49.3–61.8 (56.0) %
Number (and %) of variable sites in all taxa	189 (68.7 %)
Intra-specific distance	0.000
Inter-specific distance (mean)	0.248–0.987 (0.468)

### Discrimination power analysis

**Fig. 1:**
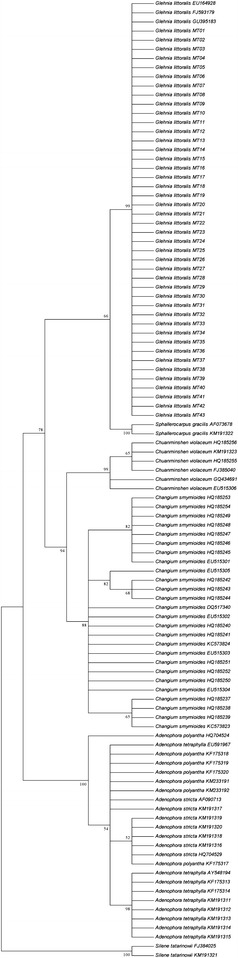
The NJ tree of *Glehniae Radix* and its adulterants, based on ITS2 sequences. Bootstrap analysis (1000 replicates) was conducted to estimate the statistical supports of the topology of the consensus tree. Bootstrap values are shown next to the branches (values below 50 % have been omitted)

**Fig. 2:**
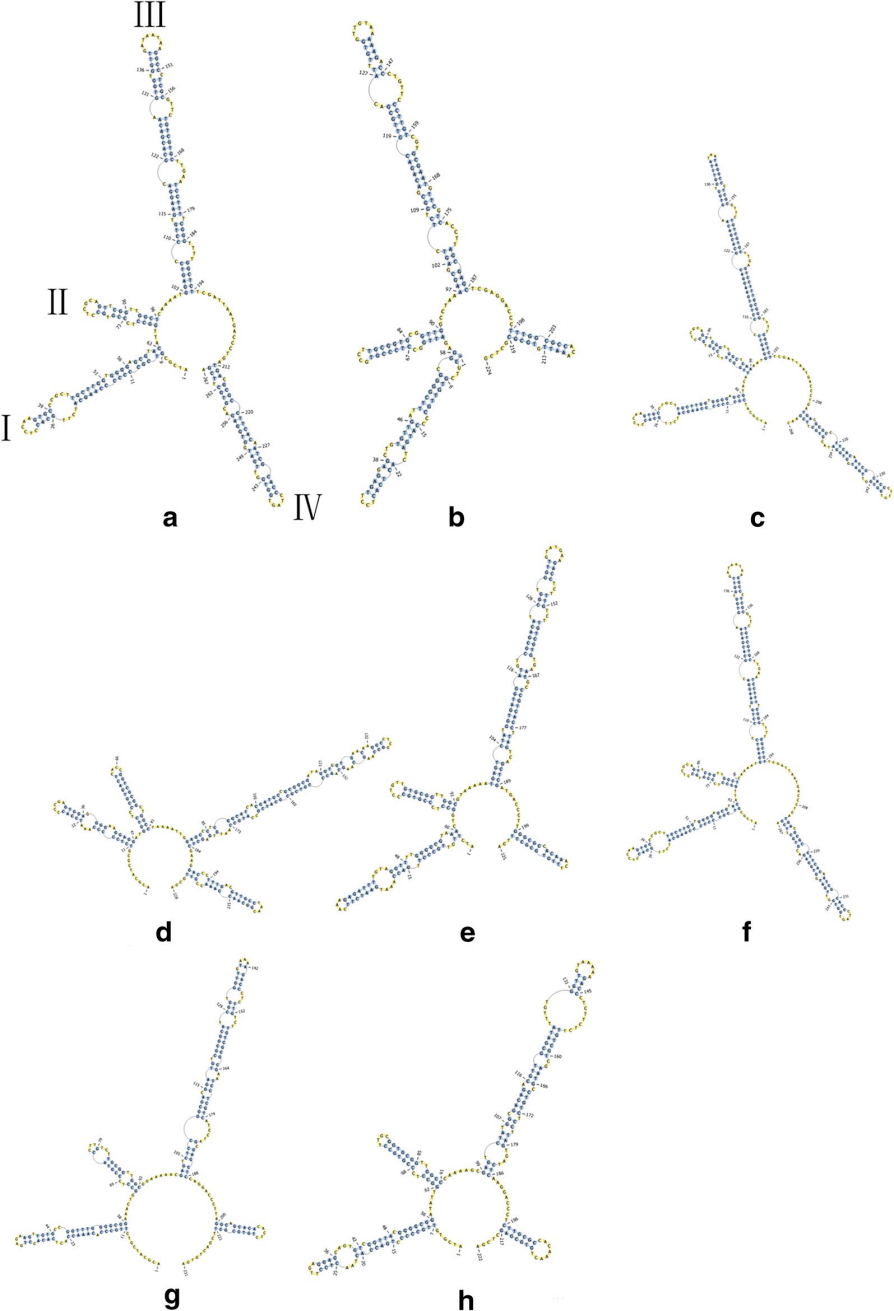
The comparison of ITS2 secondary structures in *Glehniae Radix* and its adulterants. **a** *Adenophora polyantha* HQ704524; **b** *Chuanminshen violaceum* GQ434691; **c** *Adenophora tetraphylla* AY548194; **d** *Silene tatarinowii* FJ384025; **e** *Sphallerocarpus gracilis* AF073678; **f** *Adenophora stricta* AF090713; G *G. littoralis* FJ593179; **h** *Changium smyrnioides* HQ185237

The reliability of the species identification was calculated by the BLAST1, the NJ tree technique and ITS2 sequence secondary structure determination. The ITS2 region exhibited the highest identification efficiency (100 %). Figure 1 shows the phylogenetic tree from 105 ITS2 sequences represented *Glehniae Radix* and its seven adulterant species. In the cluster dendrogram, *G. littoralis* samples from different locations were monophyletic and clustered on the same branch. Furthermore, the adulterants of different species formed distinct, nonoverlapping clades. Thus, the NJ tree of the ITS2 sequences correctly placed *Glehniae Radix* and its adulterants with high statistical support (99 %). Figure 2 indicates that the predicted secondary structures of the ITS2 sequences of *Glehniae Radix* and its adulterants each had unique molecular morphological characteristics with the stem loop size, number, position and spiral angles of the four helices.

## Discussion

Previous studies have successfully used 5S rRNA spacer domains to distinguish the original *G. littoralis* plant from its related medicinal species [10, 13] These studies extracted genomic DNA samples from leaves, but did not mention commercial *Glehniae Radix* samples. A study [31] explored the utility of ITS2 sequences for barcoding *Glehniae Radix*, their results displayed the similar trend that *Glehnia Radix* and its three adulterants could be identified by the ITS2 region. But the study included only four *Glehniae Radix* sequences and six sequences derived from three adulterant species. In this study, we used 46 and 59 ITS2 sequences from *Glehniae Radix* and its seven adulterants, respectively.

The processing procedures of *Glehniae Radix* are as following: remove the fibrous roots, stems and residual impurities, wash, slightly dry, rear blanched in boiling water, remove the skin, cut and dry. After processing, genomic DNA extracted from the roots of samples was usually severely degraded or contaminated by micro-organisms [32, 33]. Moreover, it was difficult to obtain high-quality DNA from samples enriched in polysaccharide polyphenols, fibre or other storage materials [17, 24]. In this study, we added 10 % PVP-30 powder when grinding the sample in liquid nitrogen to remove contaminants (polyphenols and polysaccharides). Subsequently, we added nuclear separation liquid to the plant powder 2–3 times, and extended water bath time to ensure DNA could be fully released into the extraction buffer. Additionally, root herbs often contain soil fungi; therefore, to avoid the influence of fungal contamination, one should clean the herb’s surface and isolate the interior material during the sampling procedure. We successfully extracted genomic DNA from 59 various herb samples, including 38 commercial crude drugs and 16 dried root samples.

Because of the DNA degradation of herbal medicines, DNA barcodes that are favourable for some plant species such as *rbcL* and *matK* cannot be used to identify commercial herbs [34]. In our study, the DNA isolated from the samples was severely degraded; however, 59 different versions of the ITS2 barcodes were readily retrievable due to the short length of the ITS2 in these samples (202–266 bp). Shorter fragments are easier to amplify from herbarium DNA [35], and the length of the ITS2 region might be sufficient to allow amplification without high-quality DNA [19, 24, 32]. Although multiple sequences were detected from a single individual due to its multicopy genes [36], intragenomic ITS2 variation typically occurred at only a very few, extremely variable, positions; thus, the ITS2 region can be treated as a single gene [37–39]. In our study, 46 sequences of the *Glehniae Radix* ITS2 barcode were obtained and were identical to each other. Xin et al. [40] also recognized that the application of the ITS2 barcode was not affected by the presence of multiple copies in Goji.

A successful barcode sequence requires both low intraspecific variation and high interspecific divergence. The 46 samples of *G. littoralis* analysed in this study, which were from original plants and commercial crude drugs, were representatives selected from extensive distribution areas, and their characteristics varied. The ITS2 sequences of *G. littoralis* within a given sample pool all belonged to the same haplotype, supporting Mizukami et al.’s assertion [41] that the genetic diversity among geographical strains of *G. littoralis* is narrow. The ITS2 intraspecific genetic distances in the medicinal *Panax* species were low [39]. Moreover, ITS2 displayed considerable interspecific divergence between *Glehniae Radix* and its adulterants. Thus, the ITS2 sequence as a barcode shows strong stability within species and high variability between species. Similar results have been found in *Flos Lonicerae Japonicae* (*Jinyinhua*) [23]. ITS2 sequences could be suitable for *Glehniae Radix* identification due to high conservation of the ITS2 regions derived from commercial crude drugs and original plant samples.

An NJ tree was constructed and compared the molecular morphological features of the ITS2 secondary sequences of *Glehniae Radix* and its adulterants. In the cluster dendrogram, each of the medicinal species was unambiguously distinguishable from each of the others. Furthermore, the secondary structure of ITS2 is considered a molecular morphological characteristic [30, 37]. This study demonstrated that ITS2 has a powerful identification capability, as the ITS2 sequences readily distinguished *Glehniae Radix* from its adulterants. DNA barcode discrimination technology has been applied to identify commercial plant products in teas [42] and *Hypericum* species [43], among others [26, 44].

## Conclusion

DNA barcoding based on ITS2 distinguished commercial processed *Glehniae Radix* from common herbal adulterants.

## Additional file

10.1186/s13020-015-0071-8 Results from PCR amplification of the ITS2 regions of G. littoralis M: marker, 1, 18 and 33: negative control (CK), 2–17, 19–32, 33–46: G. littoralis.

## Abbreviations

ITS2: The second internal transcribed spacer
NJ: neighbor-joining
ITS: internal transcribed spacer
K2P: kimura 2–parameter
CM: Chinese medicine
BOL: the Barcode of Life Plant Working Group
CBOL: The China Plant BOL Group
PVP: Polyvinyl pyrrolidone
