# Supplementary figures and images for: Authentication of commercial processed Glehniae Radix (Beishashen) by DNA barcodes
Source: Chin Med. 2015 Nov 30;10:35. doi: 10.1186/s13020-015-0071-8 (PMC4665842; doi:10.1186/s13020-015-0071-8)

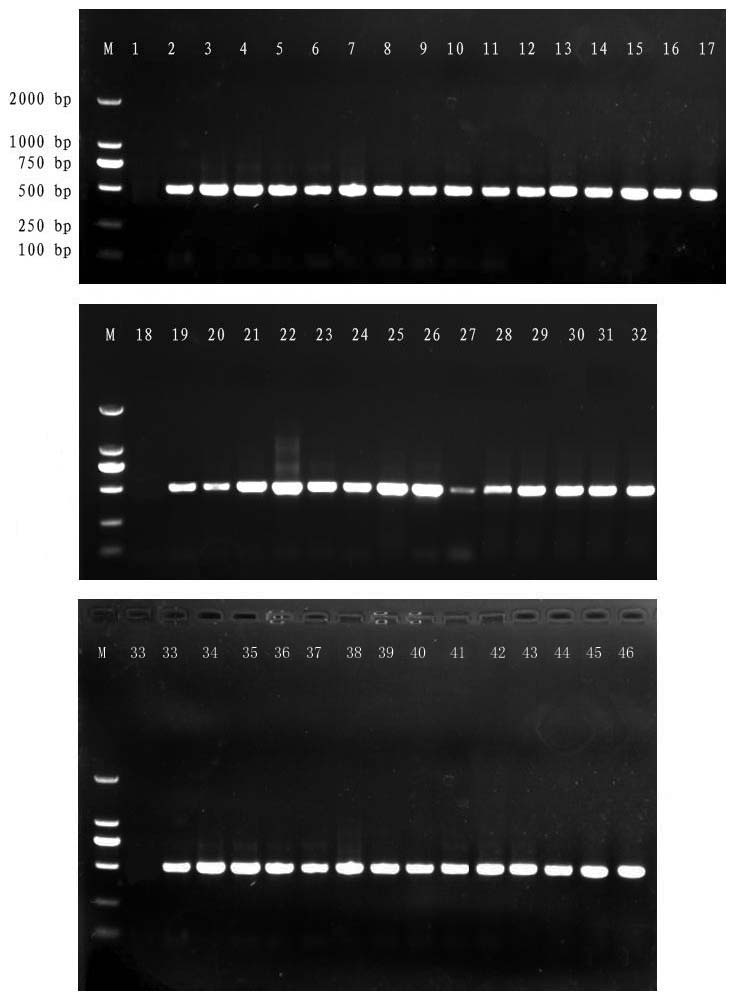

Supplement: Supplementary file 1 — 10.1186/s13020-015-0071-8 Results from PCR amplification of the ITS2 regions of G. littoralis M: marker, 1, 18 and 33: negative control (CK), 2–17, 19–32, 33–46: G. littoralis. [file 13020_2015_71_MOESM1_ESM.jpg]
